# Diagnostic puzzle of *Anopheles* species using morphological vs. molecular approach: a case study in Tripura, a hyperendemic malaria state, Northeast India

**DOI:** 10.1186/s41182-025-00747-z

**Published:** 2025-10-09

**Authors:** Ashwarya Kumari Sihag, Jadab Rajkonwar, Phiroz Gogoi, S. Vezhavendan, Savitha Chellappan, Sneha Suresh Satpute, Prantosh Malakar, Sangit Debnath, Kalpana Baruah, Harpreet Kaur, Sarala K. Subbarao, Dibya Ranjan Bhattacharyya, Ipsita Pal Bhowmick

**Affiliations:** 1 Regional Medical Research Centre, Northeast Region (RMRC-NE)-ICMR, Dibrugarh, 786001 India; 2 Department of Microbiology, Bharathidasan University, Tiruchirappalli, 620024 India; 3 National Vector Borne Diseases Control Programme, Ministry of Health & Family Welfare, Government of India, Delhi, 110054 India; 4 Indian Council of Medical Research (ICMR), Ramalingaswami Bhavan, New Delhi, 110029 India; 5 ICMR-National Institute of Malaria Research, Sector-8, Dwarka, New Delhi 110077 India

**Keywords:** *Anopheles fluviatilis*, *An. minimus*, Morphological misidentification, Molecular approach, Hypermelanism

## Abstract

The North-East India Region (NER), an area with several malaria-endemic pockets, differs from the rest of India in vector composition, with *An. fluviatilis* as the only common major vector, mostly reported during winter in NER but perennially in mainland India. However, in most cases, the most common method of morphological identification has been used, with few studies comparing both. During our longitudinal (2019–2024) entomological study in Tripura, a major malaria-contributing NER State, morphological techniques identified 9 *An. fluviatilis* collected during winter months, along with 500+ *An. minimus*. However, molecular investigations confirmed *An. fluviatilis* to be *An. minimus*, with 3 new haplotypes. Previously, *An. fluviatilis* identified morphologically and cytotaxonomically from another state of NER and was later molecularly shown to be a morphological variant of *An. minimus*. Due to hypermelanization, absence of presector pale spots on the wing and palpal banding pattern, *An. minimus* earlier probably during winter was misidentified as *An. fluviatilis* in NER while it may not be present in NER. These studies suggest that combined morphological and molecular methods should be employed for the correct identification of *Anopheles* species in NER. Morphological misidentification of NER vector as mainland vector can mislead vector control policies, causing public health havoc.

Subfamily Anophelinae of mosquitoes of the order Diptera has three genera globally, but in India, only the genus *Anopheles* is present, divided into two subgenera, *Anopheles* and *Cellia*. Species belonging to the subgenus *Cellia*, which contains most of the important vectors of human malaria in India, are placed under several taxonomic categories called series. One such series, the Myzomyia series, is now represented by 13 species in the Oriental region [1, 2]. The seven species included in India under the Myzomyia series are *An. majidi*, *An. jeyporiensis*, *An. aconitus*, *An. varuna*,* An. culicifacies*, *An. fluviatilis*, and *An. minimus*.

In India, the North-East India Region (NER), a major malaria-contributing region, is different from the rest of mainland India in several aspects, with a major distinguishing factor being the vector composition. *An. culicifacies*, *An. stephensi* and *An. fluviatilis* are major primary vectors of mainland India [3], whereas *An. baimaii* and *An. minimus* are reported as major primary vectors in NER [4], with only one reported as common major vector, *An. fluviatilis*. Mostly reported during winter months, it was considered a relay transmitter continuing transmission during the dwindling density of *An. minimus* during winter [5]. It was reported from Assam state in NER as early as 1902 [6] and 1923 [7]. *Plasmodium* infectivity by detecting sporozoites in the salivary gland via dissection was reported in Assam and Arunachal Pradesh [6–8]. However, these studies mainly employed only morphological identification methods, the most common method of identification before the advent of molecular tools and still widely used, with few studies involving both methods.

*An. fluviatilis* found in Assam*,* identified morphologically and cytotaxonomically, was later shown to be a morphological variant of *An. minimus* by molecular studies [9]. Even though *An. fluviatilis* has been considered among the three primary vectors in NER, its role in Tripura, a state of NER, is not yet shown, except for a solitary report of its presence in the 1950s [10]. Few entomological studies have been reported in Tripura, but no longitudinal studies have been conducted earlier. Tripura is critical from a malaria perspective as it contributes disproportionately high malaria cases, e.g., with ~ 0.3% of India’s population contributing to ~ 10% of malaria cases in 2023 (National Center for Vector Borne Diseases Control, NCVBDC). Hence, with India’s goal of 2030 Malaria elimination, it is imperative to map Tripura’s vectors properly.

Hence, we conducted a longitudinal monthly entomological study based on the malaria incidence using CDC light traps between dusk and dawn in the 11 hamlets from 4 high malaria-endemic subcenters, Gurudhan, Shikharibari, Maldapara and Karnamani of the Dhalai, the highest malaria-affected district of Tripura, from May 2019 to April 2024 (Fig. 2).

During this study, we collected 504 specimens morphologically identified as *An. minimus*. Another nine specimens of *Anopheles* were also collected, which were first identified using the standard keys of Das and Rajagopal [11] with morphological characteristics, especially in their maxillary palpi and wings that resemble *An. fluviatilis* (Fig. 1) (Table 1). The palpal banding pattern and presector pale spot at the base of the wing are the two most distinguishing characters to identify *An. minimus* [12]. These specimens were collected from four hamlets during December 2021 and 2023, and March 2024.

**Fig. 1:**
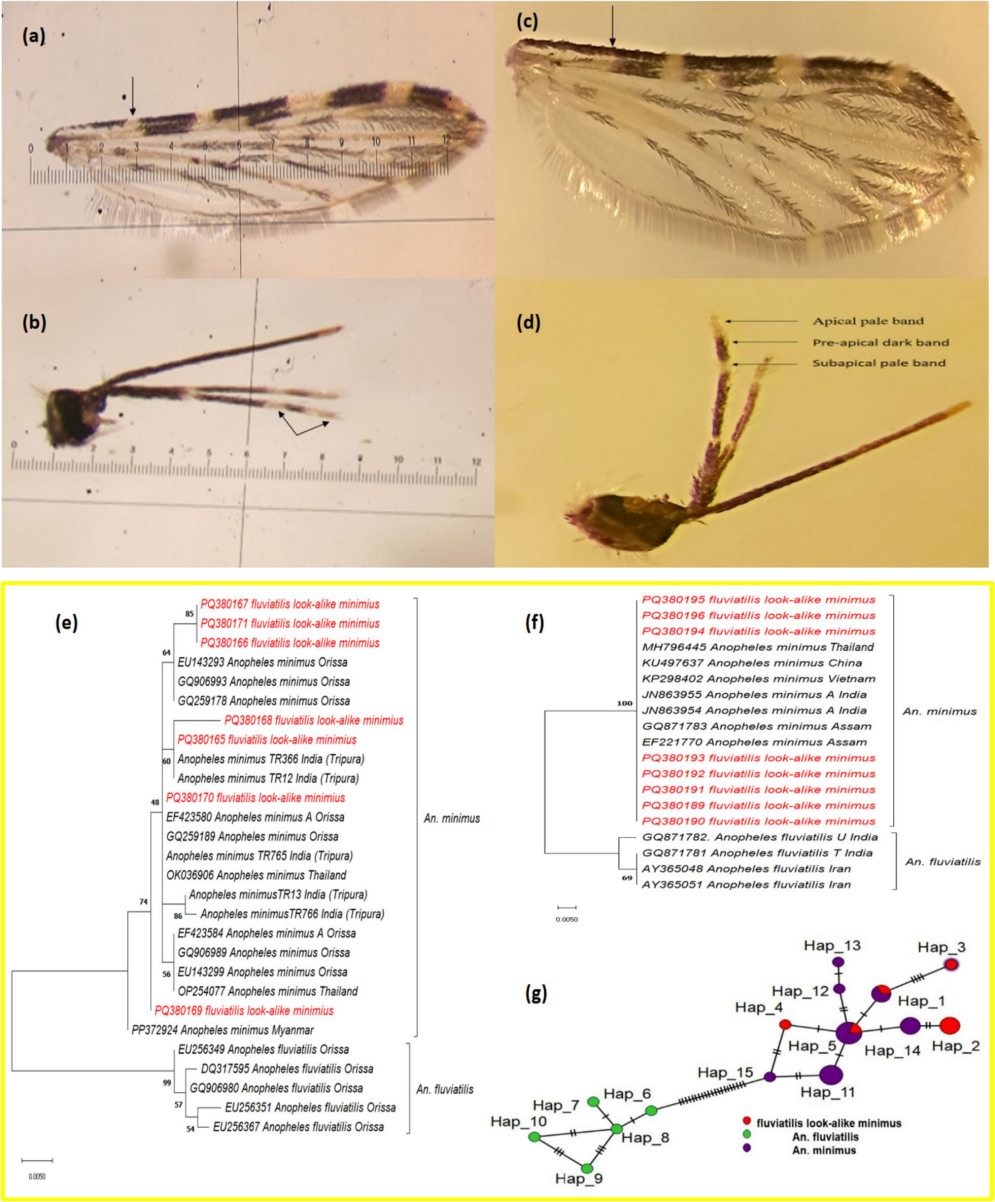
**a** Wing of a typical *An. minimus* showing the presector pale spot in the costa, **b** palpi of a typical *An. minimus* with both apical and subapical pale bands mostly equal and dark band in between equal or smaller, **c** wing of a morphovariant *An. minimus* showing lack of presector pale spot in the costa, **d** palpi of a female morphovariant, *An. minimus* with small apical and subapical pale bands and longer preapical dark bands; maximum likelihood phylogenetic tree of the *fluviatilis* look-alike *minimus* **e** ITS2 gene sequence, created by applying the Juke Cantor parameter with a bootstrap value of 500, **f** CO1 gene sequence, created by applying the Tamura 3 parameter with a bootstrap value of 500, **g** a haplotype network for the *fluviatilis* look-alike *minimus* sequence was constructed via a minimum spanning network approach. Isolates are distinguished by color, reflecting the species and the haplotypes identified in the present study are illustrated in red (*fluviatilis* look-alike *minimus*), green (*An. fluviatilis)* and purple (*An. minimus*)

**Table 1:** Key morphological characters to differentiate adult females of *An. minimus* and *An. fluviatilis*

Characters present in	*An. minimus*	*An. fluviatilis*
Maxillary palpi	Apical pale band of female palpi is almost equal to the subapical pale band. The dark band in between is mostly equal, shorter or slightly longer than the pale bands	Subapical pale band is shorter than the apical pale band. The dark band in between is much longer than the subapical pale band
Wing	Presence of presector pale spot in costa at least one of the wing	Presector pale spot absent in costa

*Note: An. minimus* and *An. fluviatilis* cannot be differentiated in larval and pupal stages

Except *An. culicifacies, An. jeyporiensis* and *An. majidi,* most of the Oriental Myzomyia series *Anopheles* are most difficult to identify in the adult stage because of overlapping characters [1]. But most of these species, except *An. fluviatilis* and *An. minimus*, others possess primary identifying characters in their larval and pupal stages [1]. Because of variations, *An. minimus* reported to resemble *An. aconitus, An. varuna* and *An. fluviatilis,* including many intermediate forms [1].

Hypermelanism is the increase of dark pigment melanin, resulting in dark coloration in insect bodies. Hypermelanism can create problems for species identification, especially in closely related species. For example, the presence of presector pale spots on the costa in the wing is an important characteristic to identify *An. minimus.* But during winter, because of melanization, these presector pale spots become dark, leading to misidentification.

Studies on biotic and abiotic factors capable of influencing melanization were unavailable in mosquitoes. But studies carried out in the laboratory in other insect species such as scavenger fly species (*Sepsis thoracica*), suggested that developmental temperature and larval density can influence the activity of a key enzyme call phenoloxidase essential for insect pigmentation [13]. Harrison [1] also remarked earlier that frequency of presector pale spots on costa in the wing of *An. minimus* is probably influenced by temperature and immature habitat.

In our present study spanning May 2019 to April 2024, we collected 504 typical *An. minimus* morphologically, out of which 395 were confirmed *An. minimus* by molecular identification and nine specimens identified as *An. fluviatilis* by morphological method but turned out to be *An. minimus* by molecular method having *An. fluviatilis* like morphological characters. Although the overall percentage of such specimens is less, if we consider the collection made in cooler months as *An. fluviatilis* is known to appear in winter, then percentage will increase. However, the important question is the identity of such species, more than its density. It is because *An. fluviatilis* is a major vector of human malaria in the country and northeast India, this species was considered among the three most important vectors along with *An. baimaii* and *An. minimus* [10]*.* Even in Tripura, the presence of this species was reported in the 50s. Member species of the Myzomyia Series are known for their close similarities as well as for variation of morphological characters [1]. Although there were many studies on this aspect in other countries [1], such studies especially in *An. minimus* was scarce in India mainly for two reasons. First, Christophers [12] considered some characters like presector pale spots on costa in the wings of *An. minimus* to be most constant and two dark spots in the wing vein Cu1 distal to m-cu cross vein as rarely variable. However, he considered palpal banding to be variable. The second factor is that among the members of Myzomyia Series like *An. aconitus*, *An. varuna*, *An. jeyporiensis*, *An. culicifacies* and *An. minimus* variations in adult morphology can be verified by doing a larval survey in the locality. Because in these species confirm identity is possible in immature stages. However, the same is not possible between *An. fluviatilis* and *An. minimus* as differences are lacking in the differentiation of these two species in their immature stages. Because of such reasons, *An. minimus* identified as *An. fluviatilis* in northeast India, and this could be resolved only after the advent of molecular methods. Now, it is becoming important to clarify the identity of such species.

As our specimens resembled *An. fluviatilis,* considered as mainland India’s primary vector, it was necessary to confirm the actual species status to determine whether they are *An. fluviatilis* or variation of other prevalent species. Hence, we subjected all our morphologically identified *An. fluviatilis* specimens for molecular studies using methods described earlier [14]. Briefly, the mosquito specimens were dissected, and extraction of genomic DNA was performed via the QiAmp DNA Extraction Kit (Qiagen). For species confirmation, both the CO1 [15] and ITS2 [16] genes were amplified and sequenced on an Applied Biosystem 3500 Genetic Analyser. The CO1 and ITS2 sequences were BLAST searched in the NCBI database to retrieve homologous sequences from GenBank. MEGA X version 10.2.6 was used to generate maximum likelihood phylogenetic trees, and DnaSP [17] and PopART v.1.7 [18] software was used for creating haplotype network analysis via the CO1 gene. The specimens were confirmed to be *An. minimus* (Fig. 1)*.* The geographical distribution map was prepared using QGIS 3.34 software, and the analysis is aided by the download of base maps from India’s survey (Fig. 2).

**Fig. 2:**
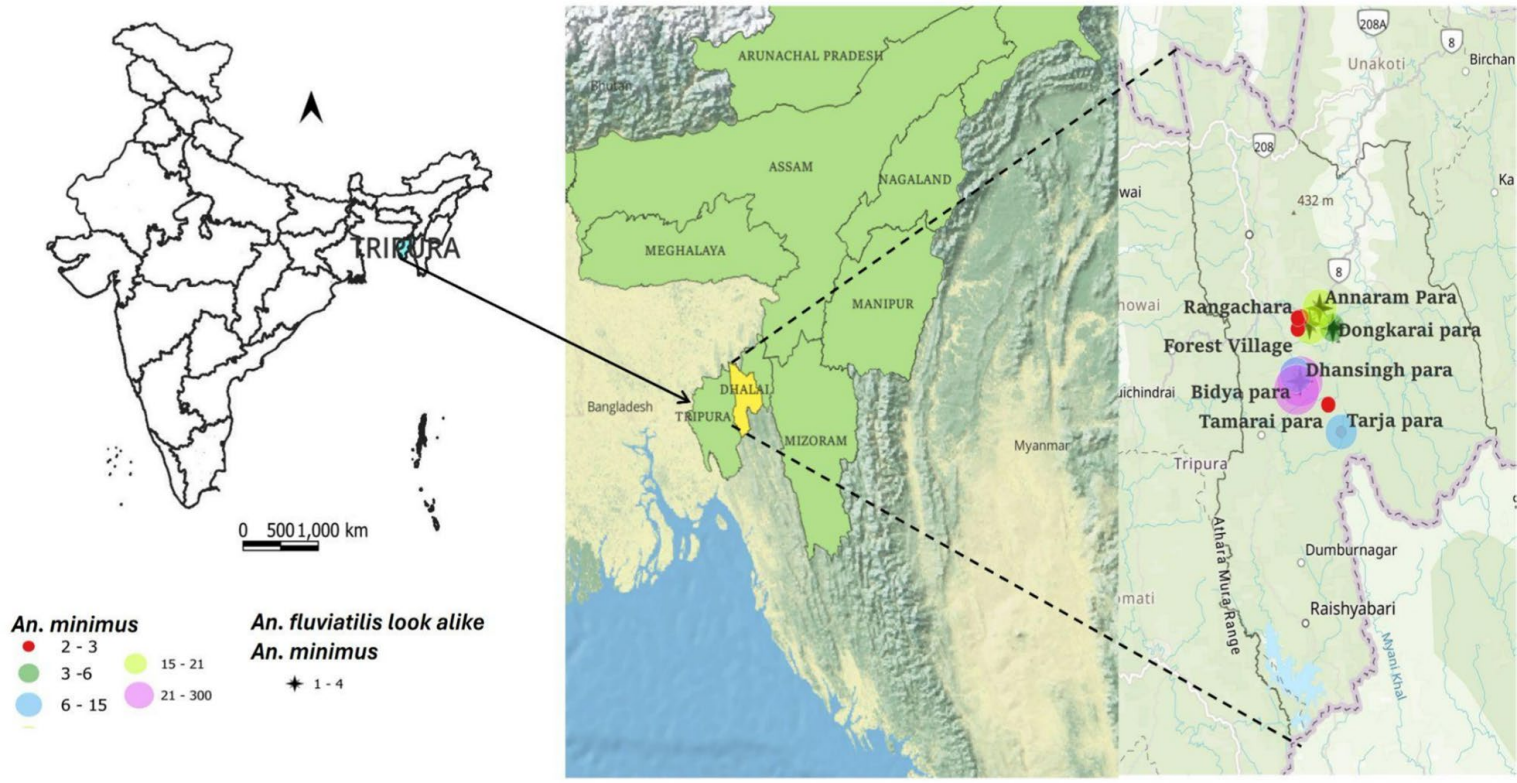
Map of Dhalai district showing our study sites from where collection of *An. minimus* and *fluviatilis* look-alike *minimus*, later molecularly confirmed as *An. minimus* was done from the year 2019–2024. Due to very close proximity among the hamlets, 3 villages, Khusidhan, Ananta-Mainyaram 1 and 2 are not visible

Among the nine samples collected, however, few samples failed to amplify, six were sequenced for CO1 (accession nos. PQ380165–PQ380170), and seven for ITS2 (accession nos. PQ380189–PQ380196), and phylogenetic trees were constructed separately for each gene, which included sequences of *An. minimus* and *An. fluviatilis* from other states and countries. They most closely matched the *An. minimus* ITS2 sequences of Tripura, Assam, Thailand, China, and Vietnam (Fig. 1e) and the *An. minimus* CO1 sequences of Odisha, Tripura, and Thailand (Fig. 1f). The *An. fluviatilis* node of the CO1 tree was supported by a bootstrap value of 99, whereas the *An. minimus* node of the ITS2 tree was supported by a bootstrap value of 100. Additionally, haplotype analysis revealed that *An. minimus* and *fluviatilis* look-alike *minimus* presented the same haplotype (Fig. 1g) with the report of 3 new haplotypes. Also, out of these nine samples tested for *Plasmodium* positivity by methods described earlier [14], one was positive for *Pv* and one for mixed (*Pf* + *Pv*). If only it had morphological identification, *An. fluviatilis* would have been reported as a new vector emerging from the malaria-endemic region of Tripura, but our molecular studies confirm that these are variations of *An. minimus* only.

Earlier, cytotaxonomic studies, which were carried out on *An. fluviatilis* from the Kamrup district of Assam identified these samples as *An. fluviatilis* U (of the three sibling species identified S, T and U) [19] based on the banding pattern of polytene chromosome arm 2 [20]. With the combination of cytotaxonomic and molecular methods, it was confirmed later that it was *An. minimus* [9]. Because the appearance of the mosquito specimens was usually low in density, especially in the winter months, with a lack of presector pale spots in the wing and a palpal banding pattern, entomologists probably identified them as *An. fluviatilis*. With the advent of molecular tools, we now conclude that these samples were hypermelanic variants of *An. minimus*.

In the China museum, the specimens were identified as *An. fluviatilis* because palpal banding patterns which were later confirmed to be *An. minimus* s.l. via molecular method [21]. In Vietnam, *An. varuna,* a non-vector species, was misidentified as an unusual morphotype of *An. minimus* and targeted to control as a vector [22], suggesting that *Anopheles* species belonging to the Myzomyia series are highly variable in the Oriental region. The situation becomes complex when a species resembles a primary vector due to variation, such as in NER. It would be worthwhile to explore the biological basis and implications of these variations in the future.

Due to hypermelanization, lack of presector pale spots in the wing and a palpal banding pattern, entomologists probably misidentified *An. minimus* in winter as *An. fluviatilis* in NER and it is now safe to conclude that these previously reported *An. fluviatilis* samples from NER were actually hypermelanic variants of *An. minimus,* with the former having an unlikely existence in NER.

Overall, our study concludes that any reports of the appearance of *An. fluviatilis* at NER or identification of closely related species especially in the winter months, should be confirmed via molecular methods as morphological identification can lead to misidentification and mislead vector control policies in NER, which has some of the bottleneck regions for malaria elimination in India. Hence, it is very important to correctly identify the vectors here.

## Data Availability

The genome sequences in this study were deposited in the NCBI database under accession numbers PQ380165–PQ380170 and PQ380189–PQ380196.
